# Clinical Review, and Hospital Reports

**Published:** 1843-04-01

**Authors:** 


### Clinical Review.

### King's College Hospital Report for 1842. With Remarks. By William Augustus Guy, M.B.*

During the year 1842, 1383 patients were admitted into King's College Hospital; of this number, which, however, is exclusive of casualties, and includes 180 midwifery cases, 93 died, being as nearly as possible 1 in 15, or 6·72 per cent.

On examining the number of the several diseases which occurred in the several months and quarters of the year 1842, and which will present a tolerably correct view of the sickness prevalent in the central districts of the metropolis, it appears that the class of febrile affections, the contagious exanthemata, catarrh, diseases of the organs of digestion, and the mixed group, including struma, gout, plethora, &c. resemble each other in the order of frequency, the greatest number of cases of each class occurring in the third, and the least in the first, quarter; and that the quarters come in the following order, beginning with that in which the fewest cases occurred;—*first, fourth, second, third.*

The affections of the organs of respiration follow the inverse order of the classes just mentioned, the quarters appearing in the following order;—*third, second, fourth, first.* Rheumatic affections, and diseases of the urinary organs follow in the order 1, 2, 3, 4; diseases of the nervous system, of the skin, and those of females, appear in the order 1, 4, 3, 2; those of the circulating system are represented by 1, 2, 4, 3; and this is the order of the syphilitic disorders.

On looking to the general total of all diseases, the first quarter is found to be the most healthy, then the last quarter, whilst the second and third present the greatest amount of sickness. The months come in the following order, beginning, as before, with the most healthy,—January, March, April, December, February, November, October, May, July, September, June, August. The amount of sickness, however, by no means corresponds with the mortality, as will be seen by referring to the Registrar-General's table of mortality for the Metropolis for 1842, which will be found in another part of this Journal.

Remarks.—*Febris Continua.*—But few cases of continued fever appear in this or in the two preceding reports. In fact, 1840, 1841, and 1842 have been remarkably free from fever. There is, however, every indication of an approaching epidemic in the present year.

*Infantile Remittent Fever.*—Cases of this were greatly in excess during the third quarter; two-fifths of the number occurring in the months of July, August, and September.

*Catarrh.*—This could scarcely be said to have been epidemic during the past year; the numbers in the several quarters being nearly the same. In 1841, the proportion of cases of catarrh and influenza to the total number of patients, was as 1 to 19—in 1842, as 1 to 27.

*Muscular Rheumatism.*—This disease was most prevalent in the last two quarters of the year. The larger number occurred in December, after a sudden change from severe cold to the mild temperature of Spring.

---

* Medical Gazette, March 10th.

*Spinal Irritation, Pleurodynia, Muscular Pain of the Abdomen.*—These include many cases of what are usually termed hysteria. Spinal tenderness is generally accompanied by pain in the abdominal parietes, more rarely by acute pleurodynia, and in other cases by pain in both these situations; the pain is often so acute as to make the patient cry. Perhaps there is no suffering which so often makes the patient cry as that produced by muscular pain. As a matter of diagnosis, this fact is worth remembering, as pain connected with more severe disease rarely expresses itself by tears. Whenever a female presents herself in tears, complaining of acute pain in the chest or abdomen, the spine should be examined, when pain will generally be discovered about the dorsal region of the vertebral column. On striking this, pain will be felt not only in the spine, but darting towards the affected muscle. When cough is present, it will be produced in like manner by striking the tender portions of the spine.

*Phthisis.*—A marked disproportion in the number of cases of this disease occurring in the two sexes, was seen in this, as in the preceding year. For the three years, the total numbers were—males 650, females 302, or 7.12 per cent. for males; 2.95 for females, and 4.92 for both sexes. Consequently the proportion of males to females is as about 7 to 3. Or, in males, consumptive cases form about a fourteenth of the total number of cases; in females, about a twenty-third; and in the two sexes together, about a twentieth.

*Mimosis Inquieta.*—This is a term used to express that restless, uneasy, nervous state into which females are apt to be thrown by sudden shocks, by long-continued anxiety, or by slow drains upon the system. The principal causes are, fright, anxiety of mind, suppression of the menstrual discharge, debilitating discharges, &c. It is also frequently present during convalescence from debilitating causes. The treatment consists in a combination of tonics and sedatives.

---

### SOME CASES OF SUPPURATION IN UNUSUAL SITUATIONS. By HENRY JAMES JOHNSON, Assistant-Surgeon to St. George's Hospital.

There is nothing very striking in the following cases, but, perhaps, they may be considered not entirely devoid of practical interest, for no inconsiderable portion of the duty of a surgeon consists in the detection and discharge of confined matter.

CASE 1.—*Abscess behind the Mamma—Puncture—Cure.*

I have already related the particulars of this case at the Westminster Medical Society, and it was subsequently reported in the Lancet, and quoted in a late number of this Journal. I shall merely refer to the main points.

The patient was a middle-aged woman, the wife of a poor tradesman, who had long laboured under dyspepsia and an indifferent state of health. She was emaciated, and in that condition in which malignant disease might be supposed likely to occur. The menstrual discharges were regular, and she had been a mother some months prior to my seeing her.

She applied to me with a painful swelling of the left breast, which she supposed was cancer. Several surgeons had seen it, and had told her that it was a tumor of the breast which would probably be serious. On examining the breast carefully, it was found to be slightly enlarged, hard, and nodulated; manipulation gave but little pain, but she complained of occasional lancinating pains shooting through the gland, somewhat resembling those which accompany the progress of scirrhus. There was no retraction of the nipple. There was an obscure sense of fluctuation, but whatever the fluid might be, it was very deep-seated. I ordered some leeches and prescribed some medicines, which I need not mention.

In three weeks, the tumor had slightly increased in size, was more tender upon pressure, and communicated a rather more distinct, though still very obscure sensation of fluctuation. I thought that it might be a deep-seated abscess, and plunged a grooved-needle through the substance of the breast; the needle, however, was wiped clean by the mamma, and furnished no satisfactory indication of any kind. Still convinced that there was deep-seated matter, I thrust in a lancet, when a large quantity of pus issued, revealing an abscess in the loose cellular texture, between the mammary gland and the pectoral muscle. A piece of lint was introduced into the wound, and the orifice kept patent, by the uniting edges being occasionally forcibly torn asunder; a method which is a valuable one to employ in cases such as this, as in time a mucous membrane is formed, which lines the aperture, and prevents its closing. I prescribed tonics, and sustained the general health. I found one aperture insufficient to evacuate the abscess thoroughly, and was under the necessity of making another and a dependent counter-opening. Both remained patent for some time, then closed, the depressed scars subsequently shewing the mamma adherent to the subjacent textures.

This is not uninstructive. Two other cases of abscess behind the mamma have been related, in one of which the gland was extirpated, and in the other the result was unfortunate. In neither, I believe, was the real nature of the affection recognised in time.

I have twice seen the mamma removed for chronic abscess in its substance. The disease was, of course, mistaken for scirrhus. And circumscribed suppuration in the centre of the mamma, or behind it, might readily be confounded with solid tumor of the breast by one who made a hasty or a superficial examination.

Case 2.—*Abscess beneath the Pectoral Muscle, pointing in the Axilla and beneath the Clavicle.*

A mother brought me her infant, about ten months old, as an out-patient at the Hospital. The child looked ill, but was not greatly emaciated, though feverish and evidently suffering pain.

There was a swelling in the axilla, tense, without discolouration of the skin, and communicating a not very distinct sense of fluctuation. The pectoral muscle, about an inch below the clavicle, was thrown forward, and also felt tense, and as if there were fluid beneath it. The swelling had formed gradually, and existed for two or three weeks. The mother could assign no cause for it.

The case appearing to be one of abscess beneath the pectoral muscle, pointing principally in the axilla, I opened it freely with a lancet, in the latter situation, concluding that the aperture there would evacuate the cavity. But in this I was disappointed. In spite of every precaution, and of very free divisions of the integument and axillary fascia, &c., the wound *would* contract, and matter collected beneath the muscle. I determined to introduce a probe from the axilla beneath the muscle, cut down upon its point, and then draw a seton from the one opening to the other. But the point of the probe was placed too deeply on the front of the chest to justify this measure. Disappointed in this, I passed a stick of lunar caustic deeply into the axillary wound, and moved it about freely to secure a free exit for the matter. The effect was contrary to what was contemplated, for the irritation of the caustic fortunately led to some fresh inflammation. The part of the suppurating cavity beneath the pectoral muscle became shut off from the axillary portion, a *distinct* abscess formed, and rapidly advanced beneath the pectoral muscle, and in the course of a week it pointed sufficiently to be with propriety opened. After this the child did perfectly well.

The occurrence of such a collection of matter in such a situation, at so early an age is probably unusual. Indeed, I have never seen an abscess beneath the pectoral muscle, independently of injury in another instance. It might naturally have been supposed that a dependent opening in the axilla would be sufficient. The result proved otherwise.

CASE 3.—*Congenital Abscess on the side of the Neck.*

Another female, brought me her child, three or four months old, on account of a large swelling on the right side of her neck. It occupied the space from the clavicle to the mastoid process, and from the median line to beyond the border of the trapezius. The skin was undiscoloured, the tumor rather painful, and it felt elastic, as from fluid in it. The child's health was tolerable, there was little pyrexia, but it seemed much distressed. The mother said that the swelling had existed from birth, but had latterly increased, and interfered with breathing.

I made a puncture with a grooved needle, and found pus and blood in it. I then made a pretty free opening with a bistoury, and discharged about three ounces of pus. The child shortly got well.

The case looked at first like one of aqueous cyst in the neck. The history favoured the idea. But there was no transparency, and the puncture with the grooved needle decided the matter. For the pus was thick, and, in all respects, that of a chronic abscess.

CASE 4.—*Neglected Bubo—Suppuration, proceeding in the course of the Deep Femoral Artery—Opening beneath the Gracilis.*

A gentleman, between 30 and 40 years of age, in the civil service of India, from which he had returned to Europe on sick-leave, applied to me on account of a swelling at the upper part of the right thigh. The front of the limb, below the ligament of Poupart, and for five or six inches downwards bulged forwards, looked globular, presented some sub-cutaneous œdema, with little reddening of the skin, but a sense of deeper fluctuation. Besides this, there were several enlarged glands in the groin. He looked ill, and many years older than he really was, a circumstance accounted for not only by his residence in India, but by habits of great excess.

It appeared that some months before he had contracted a sore on the penis. A bubo followed this, which had been opened. He had taken some mercury, and the sore had healed while the opening in the groin was nearly so, when he left Edinburgh, where he had been under treatment, and proceeded to Cheltenham. He was directed to walk and ride, although there was a good deal of fulness in the groin, and some degree of pain there. The swelling remained nearly stationary, when, about ten days before I saw him, being then in London, he contracted a gonorrhœa. For this he applied to a chemist, who gave him a very strong mixture containing capivi and other ingredients. The discharge immediately ceased, and the swelling in the thigh suddenly increased to the dimensions it presented when I saw him.

Believing that I had to do with deep-seated abscess of a formidable kind, I directed him to go to bed and foment and poultice the limb. In the course of a day or two I cut on the most prominent part, which was then nearly opposite the ligament of Poupart, and discharged about a quarter of a pint of matter. Another fluctuating point soon presented itself about the pubes. This I cut upon also, and found that it communicated with the former. But still there was deep-seated swelling, pain, and, I thought, fluctuation in the limb. I therefore cut on the fascia lata about two inches below the fold of the groin, and cautiously laid it open by a crucial incision. It was satisfactory to find that matter welled up freely from the interior of the thigh, apparently through the channel that the profunda femoris traverses. But although the discharge was now profuse, it was evident that pus was collected in the limb. It was of great size, œdematous, particularly on the inner side—and the constitutional symptoms were of a grave character. It appeared to me that, as the matter took the course of the profunda, the only chance of effectually discharging it and of saving the patient, was to cut on the inner side of the limb, behind the gracilis muscle, and turning up its border, arrive at the inclined plane of the adductor magnus, which would probably lead to the seat of the accumulation. I requested the assistance of Sir Benjamin Brodie, who kindly gave it, and supported the view that has been stated. It was carried into effect with the happiest result. After dissecting very deeply in the direction referred to, we were gratified by finding the matter ooze out from the bottom of the wound. We endeavoured at first to cut on the end of a long probe passed from the wound in the groin, but the depth of its point, and the pulsation of the femoral or the profunda felt on it, rendered this unsafe, and we fell back on the *anatomical* dissection.

It is unnecessary to pursue the details of the case. For some little time the situation of the patient was precarious, but the matter drained off, the constitution rallied, and after the lapse of two or three months, he was in a situation to remove to the country, and returned in perfect health last Autumn to India.

Any comments on this case would be superfluous, as the facts speak for themselves. But I may be permitted to observe, that it seems to offer another warning against that indiscriminate treatment of gonorrhœa during its inflammatory stage by stimulants, unfortunately too generally adopted. Not a year elapses without my witnessing the worst results from what I must consider unscientific, and, I am sure, is dangerous practice.

Case 5.—*Very extensive Chronic Abscess of the Hip and Thigh.*

The following case is introduced, not because there is anything peculiar in the situation of the abscess, but on account of its extent. It has never occurred to me to see one of such dimensions.

Miss J., about 30 years of age, residing at Brompton, consulted Dr. James Johnson last Autumn for her general health, which had long been indifferent, and shewed him a swelling on the right hip. By my father's wish I saw the lady, who placed herself under our joint care.

The swelling was evidently a fluctuating one. It extended from more than half-way down the thigh to the crest of the ilium, and, on tapping one extremity, a long wave was seen to run up or down the limb. In width it reached from the front of the buttock to the back, and occupied the whole outside of the thigh below. The skin was undiscoloured. There was very little tenderness or pain; nor was there much febrile excitement in the system, although there was a good deal of cough, and the condition of the lungs was unsatisfactory.

I learnt that the patient had suffered from symptoms of a phthisical character for some years, and had been confined for more than one Winter to the house. Last Summer she went to Belgium, and was laid up at Antwerp with pain in the hip and thigh, which were treated as rheumatic. The limb was not examined. Finding that she grew worse rather than better, she returned to England, and immediately sent for Dr. Johnson.

There could be no doubt that the case was one of chronic abscess, and it was determined to treat it in the manner adopted by Sir Benjamin Brodie. A moderate puncture was made with a lancet in a dependent position, and the matter was allowed to drain out as it would, under the influence of warm fomentations and poultices, unaccompanied with pressure. The quantity that issued was very great; I regret that it was not measured. After a few days the opening closed, when the contents of the sac began to accumulate. When this had gone to such an extent that the sac was again filling, a second punctnre was made, and a great deal of matter was again set free. This second puncture was gently kept open by pulling the edges asunder now and then. Re-accumulation was prevented, the sides of the sac came together and apparently grew so, for the discharge became scanty and only serous, and now for some time a mere aperture has remained, giving issue to a little almost undiscoloured serum.

This case is remarkable for the great size of the abscess, and the facility with which it was cured.

